# Effectiveness of digital personalized nursing pathway on postoperative rehabilitation in patients with non-small cell lung cancer

**DOI:** 10.3389/fmed.2026.1803994

**Published:** 2026-06-17

**Authors:** Yue Wang, Yanjun Shang, Ling Xu, Jianjian Ma, Aini Cai, Hanhan Hong

**Affiliations:** 1 Department of Pulmonary and Critical Care Medicine, Shanghai Changzheng Hospital, Naval Medical University, Shanghai, China; 2 Department of Thoracic Surgery, Shanghai Changzheng Hospital, Naval Medical University, Shanghai, China

**Keywords:** digital health, mobile health application, non-small cell lung cancer, personalized nursing, postoperative rehabilitation, quasi-experimental study

## Abstract

**Objectives:**

To evaluate the effectiveness of a digital personalized nursing pathway (D-PNP) compared with conventional nursing pathway (CNP) on postoperative rehabilitation in patients with non-small cell lung cancer (NSCLC).

**Methods:**

This quasi-experimental study was conducted at a tertiary hospital in China from January 2022 to June 2024. A total of 184 NSCLC patients undergoing surgical resection were enrolled (D-PNP: *n* = 92; CNP: *n* = 92). The D-PNP intervention included a mobile health application for risk stratification, personalized rehabilitation protocols, symptom monitoring, and remote follow-up. Primary outcomes were postoperative pulmonary complications (PPCs) and pulmonary function recovery.

**Results:**

A total of 172 patients completed the study (D-PNP: *n* = 87; CNP: *n* = 85). The D-PNP group had lower PPC rates (10.3% vs. 24.7%; risk difference: −14.4%, 95% CI: −25.2% to −3.6%; *P* = 0.013; NNT = 7). At 3 months, FEV_1_ recovery was superior in the D-PNP group (94.4% vs. 88.2% of baseline; between-group *P* = 0.018). The D-PNP group also showed better quality of life (*P* < 0.001 for selected 3-month domains), lower anxiety and depression scores (both *P* < 0.001 at 3 months), higher rehabilitation compliance (86.2% vs. 68.2%; *P* = 0.005), and a non-significant trend toward lower 30-day readmission (3.4% vs. 10.6%; Fisher’s exact *P* = 0.079).

**Conclusion:**

D-PNP was associated with reduced complications, improved functional recovery, and better rehabilitation adherence in NSCLC patients. Confirmation through randomized controlled trials is warranted.

## Graphical Abstract

**Figure:**
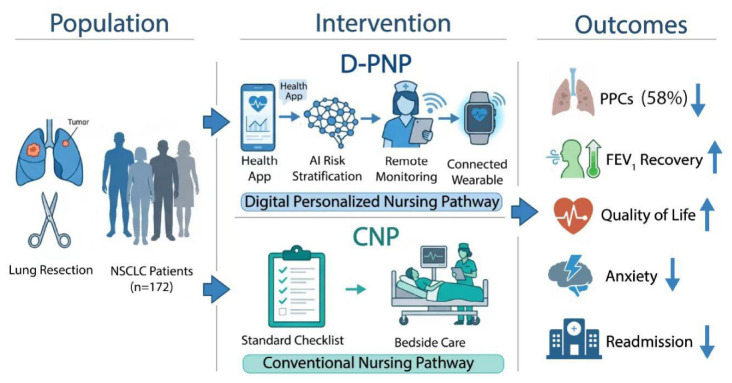
Study overview showing that, in 172 patients undergoing surgical resection for non-small cell lung cancer, the digital personalized nursing pathway (D-PNP)-integrating risk stratification, personalized rehabilitation prescription, remote symptom monitoring, and wearable activity tracking through a smartphone application-was associated with lower postoperative pulmonary complication rates, faster FEV1 recovery, improved quality of life, lower anxiety scores, and a directional trend toward lower 30-day readmission compared with the conventional nursing pathway (CNP).

## Introduction

Lung cancer remains the leading cause of cancer-related mortality worldwide, with approximately 2.48 million new cases and 1.81 million deaths reported globally in 2022 (1). Non-small cell lung cancer (NSCLC) accounts for 80–85% of all lung cancer cases, and surgical resection remains the cornerstone of curative treatment for early- and intermediate-stage disease (2, 3). With the widespread adoption of video-assisted thoracoscopic surgery (VATS) and enhanced recovery after surgery (ERAS) protocols, an increasing number of patients are now eligible for surgical intervention (4).

However, patients undergoing lung resection face substantial postoperative challenges, including pulmonary function decline, reduced exercise capacity, pain management difficulties, and risk of pulmonary complications (5). Postoperative pulmonary complications (PPCs), reported in 15–37% of cases, are significant contributors to morbidity, prolonged hospital stay, and poor prognosis (6, 7). Additionally, 30–50% of lung cancer patients experience clinically significant anxiety or depression, which can adversely affect recovery outcomes (8).

Conventional nursing pathways (CNP), while effective in standardizing care processes, often adopt a “one-size-fits-all” approach that may not adequately address the heterogeneous needs of NSCLC patients. Patient characteristics vary considerably in terms of age, comorbidities, surgical approach, baseline pulmonary function, and psychosocial factors, necessitating more individualized care strategies (9). Furthermore, the transition from hospital to home frequently results in discontinuity of care, with limited post-discharge support and monitoring.

Digital health technologies—including mobile health (mHealth) applications, wearable devices, and remote monitoring platforms—offer practical tools for postoperative nursing care (10, 11). These technologies enable real-time data collection, facilitate patient-provider communication, support risk stratification, and deliver personalized interventions regardless of geographical constraints (12). The digital personalized nursing pathway (D-PNP) integrates these technological capabilities with evidence-based nursing practices to provide tailored, continuous care throughout the perioperative journey (13).

For the purposes of this study, we operationally define the **digital personalized nursing pathway (D-PNP)** as a four-component, App-mediated nursing protocol comprising: (i) **algorithmic risk stratification** based on patient-level perioperative risk factors, (ii) **risk-tiered rehabilitation prescriptions** that match exercise volume and intensity to the assigned risk category, (iii) **bidirectional symptom telemonitoring** with rule-based alerts triggering nurse review, and (iv) **structured remote follow-up** through scheduled video consultations. The pathway is delivered through a purpose-built smartphone platform and operates across four temporal phases—preoperative, inpatient postoperative, transition (1–2 days before discharge), and home rehabilitation (3 months). A schematic of the D-PNP framework and its inputs/outputs is provided in Supplementary Figure 1.

Despite the theoretical advantages of D-PNP, empirical evidence regarding its effectiveness in NSCLC postoperative rehabilitation remains limited (10, 11). Therefore, this study aimed to evaluate the effectiveness of D-PNP compared with CNP on postoperative rehabilitation outcomes in patients with NSCLC, with specific focus on pulmonary complications, functional recovery, quality of life, psychological well-being, and healthcare utilization.

## Materials and methods

### Study design, setting, and ethical considerations

This quasi-experimental study employed a non-equivalent control group design with convenience sampling. The study was conducted at the Department of Pulmonary and Critical Care Medicine, Shanghai Changzheng Hospital, from January 2022 to June 2024. This report adheres to the Transparent Reporting of Evaluations with Nonrandomized Designs (TREND) statement for reporting standards (14).

### Participants

Patients were eligible for inclusion if they: (1) had pathologically confirmed NSCLC; (2) were clinical stage I-IIIA and scheduled for lobectomy, segmentectomy, or wedge resection; (3) were aged 18–75 years; (4) had Eastern Cooperative Oncology Group (ECOG) performance status 0–2; (5) possessed a smartphone and were capable of using mobile applications independently or with family assistance; and (6) provided written informed consent.

Exclusion criteria were: (1) concurrent malignancy or severe cardiac, hepatic, or renal dysfunction; (2) preexisting cognitive impairment or psychiatric disorders; (3) planned neoadjuvant therapy or immediate postoperative chemoradiotherapy; (4) estimated survival less than 6 months; and (5) inability to complete follow-up assessments.

### Sample size calculation

The sample size was determined *a priori* for the primary outcome of postoperative pulmonary complications (PPC). Based on previous literature in thoracic surgery cohorts and our institutional audit data (6, 7), we hypothesized a PPC rate of 28% in the CNP group and 12% in the D-PNP group, corresponding to Cohen’s effect size *h* = 0.41 (medium effect; calculated value 0.408). Because this was an exploratory quasi-experimental nursing intervention study and prior digital-health effect estimates were imprecise, a two-sided alpha of 0.10 with 80% power was prespecified for sample-size planning. Under these assumptions the required sample was 75 participants per group (PASS 15.0). After applying a pragmatic 20% inflation margin to protect against attrition, we targeted enrollment of 90 per group (total *n* = 180); 92 were enrolled per group. A sensitivity power calculation using the realized per-protocol sample (*n* = 87 D-PNP, *n* = 85 CNP) and the observed effect size (*h* = 0.385) yielded power of 0.81 at the prespecified α = 0.10 and 0.72 at the conventional α = 0.05 threshold. Accordingly, the study retained the planned power for its primary exploratory objective, while all inferential results are reported with exact P values and 95% confidence intervals.

### Group assignment

**FIGURE 1:**
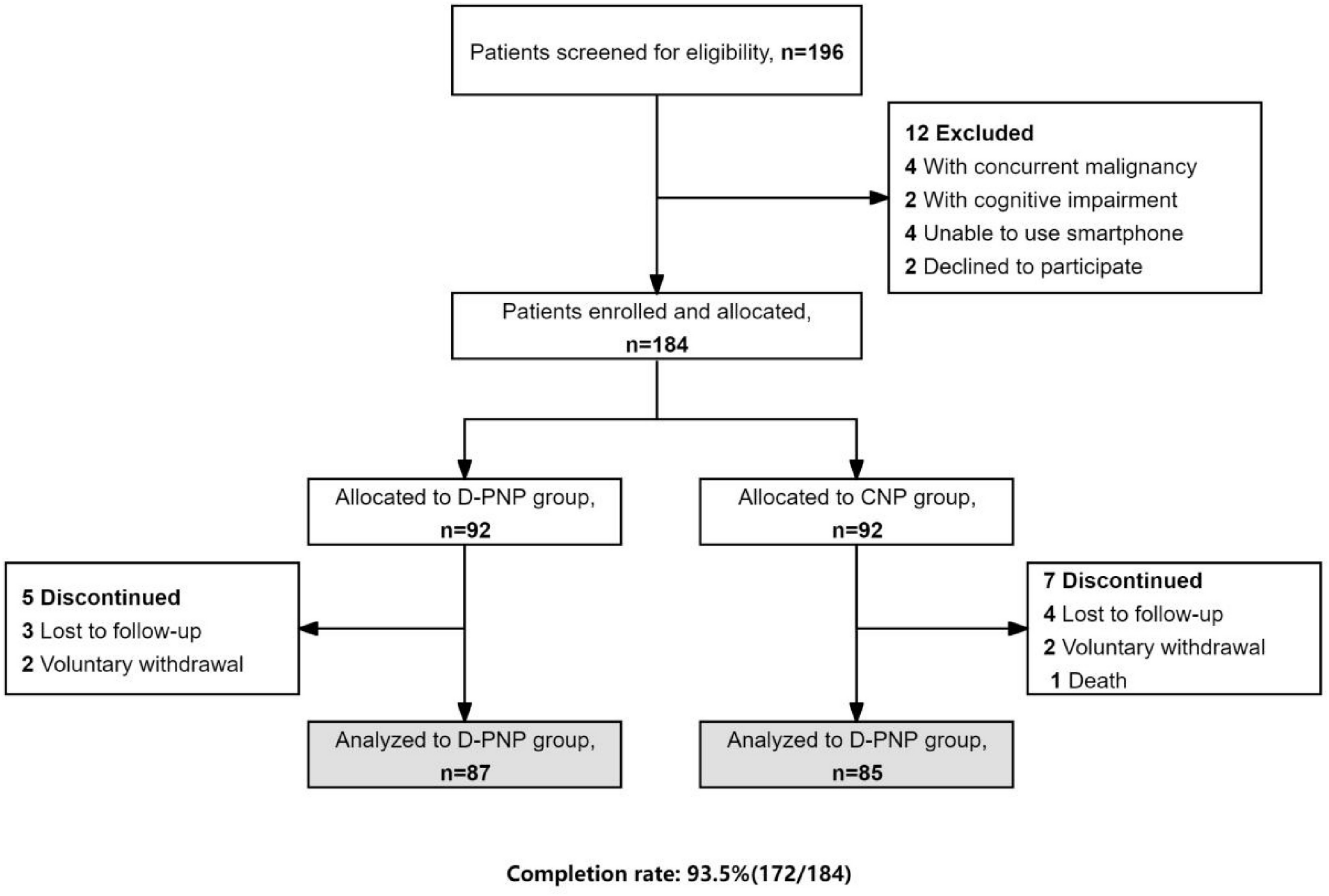
Flow diagram of participant progression through the study. Of 196 patients assessed for eligibility between January 2022 and June 2024, 12 were excluded prior to enrollment (4 concurrent malignancies, 2 cognitive impairment, 4 unable to use smartphone, 2 declined participation). The remaining 184 patients were allocated by patient preference and digital readiness to the D-PNP (*n* = 92) or CNP (*n* = 92) groups. During the 3-month follow-up, 5 patients in the D-PNP group (3 lost to follow-up, 2 voluntary withdrawal) and 7 in the CNP group (4 lost to follow-up, 2 voluntary withdrawal, 1 death from a non-study-related cause) discontinued participation. Per-protocol analysis included 172 patients (D-PNP: *n* = 87; CNP: *n* = 85), corresponding to a completion rate of 93.5%. Reasons for exclusion at each stage are shown above the corresponding box of the diagram. The flow diagram conforms to the TREND statement format for non-randomized intervention studies.

Because ethical and practical considerations precluded randomization of a technology-based intervention, we used a non-randomized concurrent control design. Patients were enrolled from January 2022 to June 2024. Among eligible participants who possessed smartphone access and were able to use mobile applications independently or with family assistance, those who agreed to receive App-supported rehabilitation were assigned to the D-PNP group, whereas those who preferred conventional follow-up without App-based monitoring were assigned to the CNP group. Patients unable to use a smartphone were excluded before allocation, as shown in Figure 1. Both groups received care from the same nursing team, who received standardized training on both protocols prior to study initiation.

### Interventions

The intervention structure is summarized below; detailed operational procedures, risk-tier prescriptions, and symptom-alert thresholds are provided in Supplementary Methods 1.

***Conventional Nursing Pathway (CNP):*** Patients in the control group received standardized perioperative nursing according to institutional protocols, including routine preoperative education, postoperative respiratory exercise guidance, early mobilization, discharge education, a printed rehabilitation booklet, and telephone follow-up at 1 week, 1 month, and 3 months after discharge. No App-based monitoring or digital rehabilitation prescription was used in this group.

***Digital Personalized Nursing Pathway (D-PNP):*** In addition to standard care, patients in the intervention group received a purpose-built mobile health application that supported patient information management, rule-based risk stratification, personalized rehabilitation prescription, daily symptom monitoring, automated nurse alerts, and remote follow-up. The intervention was delivered across four phases: preoperative preparation, inpatient postoperative rehabilitation, transition to discharge, and 3-month home rehabilitation.

***Risk Stratification Algorithm:*** The App assigned each patient a deterministic risk score (0–8) based on four perioperative domains supported by prior thoracic-surgery evidence: age, surgical approach, baseline FEV_1_% predicted, and comorbidity burden (6, 9). Total scores were mapped to low (0–2), moderate (3–5), or high (6–8) risk tiers, which determined rehabilitation intensity, ambulation targets, and symptom-alert thresholds. The scoring rule was derived from a 30-patient institutional pilot cohort and refined by expert consensus. No machine-learning component was used.

### Outcome measures

**Primary outcomes** included: (1) Postoperative pulmonary complications (PPCs), prospectively defined as atelectasis, pneumonia, pleural effusion requiring intervention, prolonged air leak ( > 5 days), or respiratory failure; and (2) Pulmonary function recovery, assessed by forced vital capacity (FVC), forced expiratory volume in 1 s (FEV_1_), and FEV_1_ percent predicted measured at baseline (T0), discharge (T1), 1 month (T2), and 3 months (T3) postoperatively.

**Secondary outcomes (exploratory)** included: (1) Health-related quality of life assessed using the European Organisation for Research and Treatment of Cancer Quality of Life Questionnaire Core 30 (EORTC QLQ-C30) (15) and lung cancer module (QLQ-LC13) (16); (2) Psychological status measured by the Hospital Anxiety and Depression Scale (HADS) (17), with scores ≥ 8 indicating clinically significant symptoms; (3) Rehabilitation compliance, assessed via app usage data (exercise completion rate, symptom recording rate) in the D-PNP group and self-report questionnaire in the CNP group; (4) Six-minute walk distance (6MWD) (18); (5) Unplanned 30-day readmission rate; and (6) Patient satisfaction with nursing services (19).

### Data acquisition and preprocessing

Patient-reported outcomes and adherence data in the D-PNP group were captured by the LungRehab App and uploaded to a hospital-managed institutional server every 24 h over an encrypted (TLS 1.2) connection. Pulmonary function (FEV_1_, FVC, FEV_1_% predicted) and 6MWD were measured by a single certified pulmonary technician on the same calibrated equipment (Jaeger Vyntus Spiro, Vyaire Medical) at baseline (T0), discharge (T1), 1 month (T2), and 3 months (T3). HADS and EORTC QLQ-C30/LC13 questionnaires were administered by a research nurse blinded to group allocation under standardized conditions. All data were double-entered into REDCap (Research Electronic Data Capture) by two independent operators; discrepancies (rate < 0.5% across the cohort) were resolved by source verification against original case-report forms. Outliers were screened using a ± 3 SD rule within group; flagged values were reviewed clinically and retained unless they reflected documented clerical errors. Non-structural missingness among common analysis variables was < 2% in the per-protocol cohort; complete-case analysis was used for variables included in each model. App-use variables were structurally unavailable in the CNP group and were therefore summarized descriptively only for D-PNP participants.

### Quality control

This study employed a single-blind design where outcome assessors were blinded to group allocation. Participants and nursing staff could not be blinded due to the nature of the intervention. All nursing staff involved in data collection received standardized training prior to study initiation. Data were independently entered by two researchers with consistency verification. Regular coordination meetings monitored study progress and data quality.

### Statistical analysis

Statistical analyses were performed using R version 4.3.3 (R Foundation for Statistical Computing, Vienna, Austria). Continuous variables were expressed as mean ± standard deviation (SD) and compared using independent *t*-tests; non-parametric Mann–Whitney U tests were used when Shapiro–Wilk testing rejected normality. Categorical variables were expressed as frequencies (percentages) and compared using Pearson chi-square tests, with Fisher’s exact test substituted when any expected cell count was < 5. Longitudinal pulmonary function outcomes (FEV_1_, FVC, and FEV_1_% predicted) measured at T0, T1, T2, and T3 were analyzed using mixed-design repeated-measures ANOVA, with group as the between-subject factor and time as the within-subject factor; Greenhouse–Geisser correction was applied when Mauchly’s sphericity assumption was violated. HADS-A and HADS-D scores, measured at T0, T2, and T3, were analyzed using the same mixed-design framework. Per-protocol analysis was the primary approach.

Effect sizes were calculated using Cohen’s *d* for continuous outcomes and risk differences (RD) with 95% confidence intervals (CI; Wald method) for binary outcomes. The number needed to treat (NNT) was calculated for the primary outcome. Given the exploratory nature of secondary outcomes, no adjustment for multiple comparisons was applied, and these results should be interpreted with caution.

To address the potential for selection bias inherent to the non-randomized assignment, reviewer-requested sensitivity analyses were conducted for the primary PPC outcome. First, propensity-score matching (PSM) used 1:1 nearest-neighbour matching without replacement on a logit-link propensity score with a caliper of 0.20 SD of the logit propensity score. The propensity model included clinically prespecified baseline factors related to both digital-intervention uptake and postoperative pulmonary risk: age, sex, smoking history, comorbidity burden, TNM stage, surgical procedure, surgical approach, and baseline FEV_1_% predicted. Covariate balance was evaluated using standardized mean differences (SMDs), with SMD < 0.10 considered acceptable. Second, multivariable logistic regression estimated the association between D-PNP and PPC after adjustment for the same covariate set. A fuller covariate model was examined only as an exploratory robustness check because the number of PPC events limited the events-per-variable ratio. The E-value was computed to quantify the strength of an unmeasured confounder required to fully explain away the observed association (20).

A two-sided *P* < 0.05 was considered statistically significant for inferential reporting; the prespecified α of 0.10 used for the *a priori* sample size calculation reflected the exploratory nature of the design.

## Results

### Participant flow and baseline characteristics

Of 196 patients screened, 12 were excluded (4 concurrent malignancies, 2 cognitive impairment, 4 unable to use smartphone, 2 declined participation), leaving 184 enrolled participants (92 per group). During follow-up, 5 patients in the D-PNP group (3 lost to follow-up, 2 voluntary withdrawal) and 7 in the CNP group (4 lost to follow-up, 2 voluntary withdrawal, 1 death from a non-study-related cause) discontinued participation. The final analysis included 172 patients (D-PNP: *n* = 87; CNP: *n* = 85), yielding a completion rate of 93.5% (Figure 1).

Baseline characteristics were comparable between groups with no statistically significant differences in age, sex, body mass index (BMI), smoking history, comorbidities, tumor stage, surgical approach, or preoperative pulmonary function (all *P* > 0.05; Table 1).

**TABLE 1:** Baseline characteristics of study participants.

Characteristic	D-PNP (*n* = 87)	CNP (*n* = 85)	t/χ^2^	*P* value
Age (years, mean ± SD)	58.34 ± 9.21	59.12 ± 8.87	0.572	0.568
Sex [*n* (%)]			0.186	0.666
Male	52 (59.8)	48 (56.5)		
Female	35 (40.2)	37 (43.5)		
BMI (kg/m^2^, mean ± SD)	23.56 ± 3.12	23.21 ± 2.98	0.762	0.447
Smoking history [*n* (%)]	41 (47.1)	38 (44.7)	0.107	0.744
Comorbidities [n (%)]				
Hypertension	28 (32.2)	31 (36.5)	0.358	0.550
Diabetes mellitus	15 (17.2)	12 (14.1)	0.319	0.572
COPD	18 (20.7)	21 (24.7)	0.405	0.524
Coronary heart disease	8 (9.2)	10 (11.8)	0.301	0.583
TNM stage [*n* (%)]			0.284	0.868
Stage I	42 (48.3)	39 (45.9)		
Stage II	31 (35.6)	33 (38.8)		
Stage IIIA	14 (16.1)	13 (15.3)		
Surgical procedure [*n* (%)]			0.156	0.925
Lobectomy	58 (66.7)	55 (64.7)		
Segmentectomy	19 (21.8)	20 (23.5)		
Wedge resection	10 (11.5)	10 (11.8)		
Surgical approach [*n* (%)]			0.092	0.762
VATS	71 (81.6)	68 (80.0)		
Open thoracotomy	16 (18.4)	17 (20.0)		
Baseline FEV_1_ (L, mean ± SD)	2.31 ± 0.52	2.28 ± 0.48	0.396	0.693
Baseline FEV_1_%pred (%, mean ± SD)	85.62 ± 12.34	84.89 ± 11.76	0.405	0.686

BMI, body mass index; COPD, chronic obstructive pulmonary disease; VATS, video-assisted thoracoscopic surgery; FEV_1_, forced expiratory volume in 1 s; FEV_1_%pred, FEV_1_ percent predicted.

### Primary outcomes

***Postoperative Pulmonary Complications:*** The overall PPC rate was significantly lower in the D-PNP group compared with the CNP group (10.3% vs. 24.7%; risk difference: −14.4%, 95% CI: −25.2% to −3.6%; χ^2^ = 6.124, *P* = 0.013). The number needed to treat (NNT) to prevent one PPC was 7. Specifically, pneumonia (4.6% vs. 14.1%, *P* = 0.034) and atelectasis (3.4% vs. 11.8%, *P* = 0.043) occurred significantly less frequently in the D-PNP group (Table 2).

**TABLE 2:** Postoperative pulmonary complications by group [*n* (%)].

Complication	D-PNP (*n* = 87)	CNP (*n* = 85)	χ^2^	*P* value
Pneumonia	4 (4.6)	12 (14.1)	4.518	0.034
Atelectasis	3 (3.4)	10 (11.8)	4.087	0.043
Pleural effusion	5 (5.7)	7 (8.2)	0.415	0.519
Prolonged air leak ( > 5 days)	2 (2.3)	4 (4.7)	0.732	0.392
Respiratory failure	0 (0.0)	2 (2.4)	—	0.241*
Total PPCs	9 (10.3)	21 (24.7)	6.124	0.013

*Fisher’s exact test. PPC, postoperative pulmonary complication. Risk difference for total PPCs: −14.4% (95% CI: −25.2% to −3.6%); NNT = 7.

***Sensitivity Analyses for Selection Bias:*** Sensitivity analyses were consistent with the primary PPC finding after adjustment for measured baseline differences. After 1:1 propensity-score matching, 79 pairs were retained from the 172-patient per-protocol cohort. All measured covariates included in the propensity model achieved acceptable post-match balance (all SMDs < 0.10). In the matched cohort, PPC occurred in 8 of 79 D-PNP patients (10.1%) and 19 of 79 CNP patients (24.1%), corresponding to an RD of −13.9% (95% CI: −25.5% to −2.4%) and an OR of 0.36 (95% CI: 0.13 to 0.93; *P* = 0.020). Multivariable logistic regression in the full per-protocol cohort, adjusting for the same clinically selected covariate set, yielded an adjusted OR for D-PNP of 0.33 (95% CI: 0.13 to 0.77; *P* = 0.013). An exploratory fuller covariate model yielded a similar estimate (OR: 0.33, 95% CI: 0.13 to 0.77; *P* = 0.014), but was interpreted cautiously because of the limited number of PPC events. The E-value for the primary unadjusted association was 4.21, indicating that an unmeasured confounder would need to be associated with both group assignment and PPC by a relative risk of at least 4.21 to fully account for the observed effect.

**FIGURE 2:**
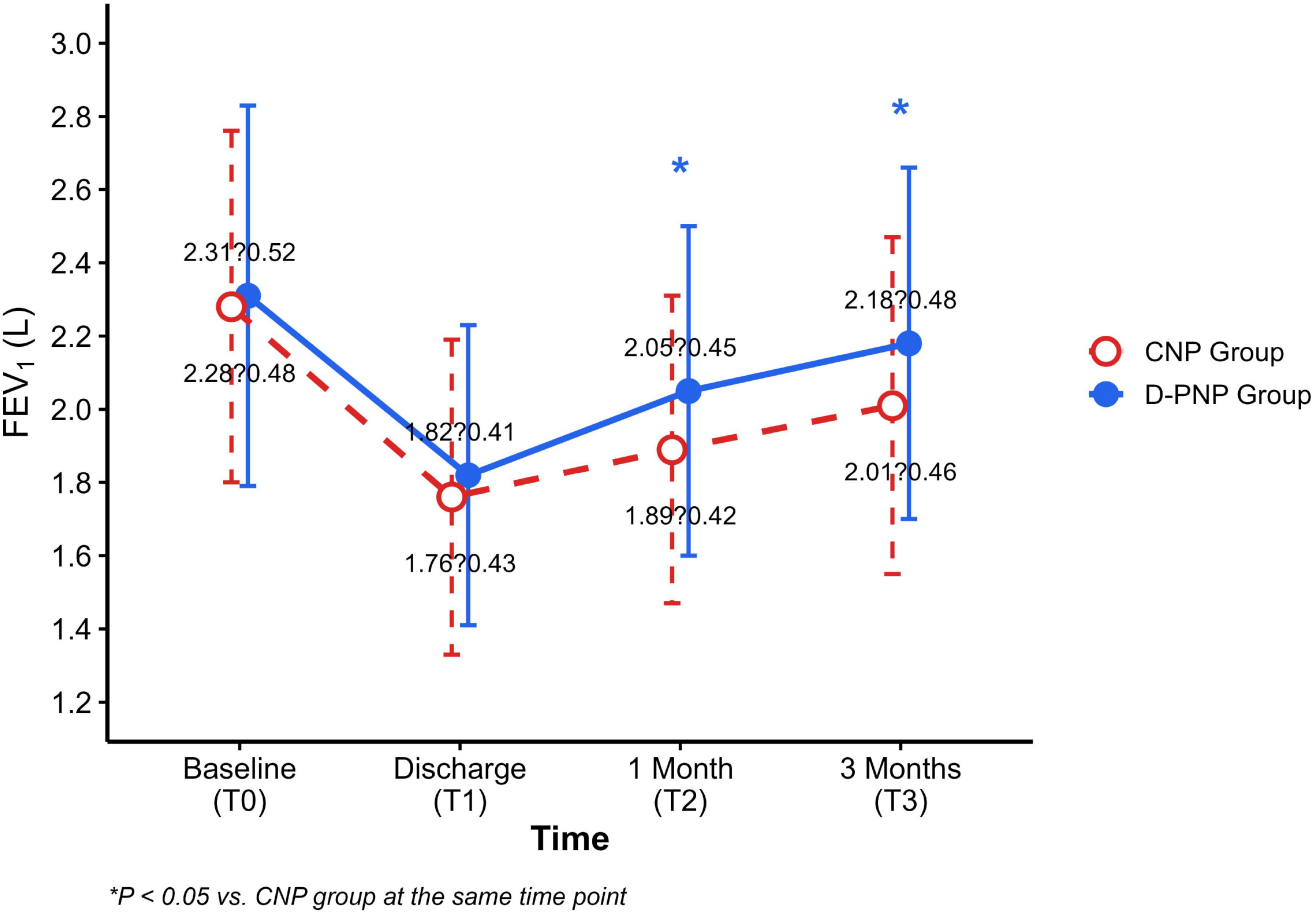
Trajectory of FEV_1_ recovery over time by study group, baseline through 3 months postoperatively. Data are presented as mean (point) ± standard deviation (error bars). The two curves represent the D-PNP and CNP groups; absolute FEV_1_ values (litres) are plotted against four sequential assessment points. Both groups showed an initial decline from baseline to discharge (T0 → T1) followed by recovery to T2 and T3. The D-PNP group recovered to 94.4% of its baseline FEV_1_ at 3 months, compared with 88.2% in the CNP group; between-group differences at T2 and T3 reached statistical significance (*P < 0.05 vs. CNP at the same time point, repeated-measures ANOVA with Bonferroni-adjusted *post-hoc* comparisons). The figure is intended to illustrate the magnitude and timing of the between-group separation, which was most evident from T2 onward. T0 = baseline (preoperative); T1 = discharge; T2 = 1 month postoperative; T3 = 3 months postoperative.

***Pulmonary Function Recovery:*** Both groups experienced an initial decline in pulmonary function following surgery, with subsequent recovery. Repeated-measures ANOVA showed significant time effects and significant between-group effects for FEV_1_, FVC, and FEV_1_%pred (all group and time effects *P* ≤ 0.003). The group-by-time interaction terms were not statistically significant (FEV_1_: *P* = 0.381; FVC: *P* = 0.352; FEV_1_%pred: *P* = 0.118), indicating that the overall recovery pattern was broadly parallel but consistently more favorable in the D-PNP group. Between-group comparisons at T2 and T3 were significant for all three pulmonary-function indices. At 3 months, FEV_1_ recovered to 94.4% of baseline in the D-PNP group versus 88.2% in the CNP group (between-group *P* = 0.018; Table 3, Figure 2).

**TABLE 3:** Comparison of pulmonary function between groups at different time points (mean ± SD).

Parameter	Group	Baseline (T0)	Discharge (T1)	1 month (T2)	3 months (T3)	F_time	F_group	F_inter
FEV_1_ (L)	D-PNP	2.31 ± 0.52	1.82 ± 0.41	2.05 ± 0.45*	2.18 ± 0.48*	37.09	9.04	1.03
	CNP	2.28 ± 0.48	1.76 ± 0.43	1.89 ± 0.42	2.01 ± 0.46	*P* < 0.001	*P* = 0.003	*P* = 0.381
FVC (L)	D-PNP	3.12 ± 0.58	2.41 ± 0.52	2.76 ± 0.54*	2.95 ± 0.56*	55.31	9.10	1.09
	CNP	3.08 ± 0.54	2.35 ± 0.48	2.58 ± 0.51	2.74 ± 0.53	*P* < 0.001	*P* < 0.003	*P* < 0.352
FEV_1_%pred (%)	D-PNP	85.62 ± 12.34	67.45 ± 10.21	76.23 ± 11.12*	81.34 ± 11.87*	86.90	13.38	1.97
	CNP	84.89 ± 11.76	65.78 ± 9.87	71.56 ± 10.34	75.67 ± 11.23	*P* < 0.001	*P* < 0.001	*P* = 0.118

*P < 0.05 compared with CNP group at the same time point. FEV_1_, forced expiratory volume in 1 s; FVC, forced vital capacity; FEV_1_%pred, FEV_1_ percent predicted; F_time, F value for time effect; F_group, F value for group effect; F_inter, F value for interaction effect.

### Secondary outcomes

Quality of life scores showed significant between-group differences at 3 months postoperatively. The D-PNP group reported higher global health status (68.44 ± 13.54 vs. 61.23 ± 12.89; mean difference [MD] 7.21, 95% CI: 3.23 to 11.19; *P* < 0.001), better physical functioning (76.85 ± 11.14 vs. 70.34 ± 10.67; MD 6.51, 95% CI: 3.23 to 9.80; *P* < 0.001), role functioning (70.05 ± 12.97 vs. 62.56 ± 12.34; MD 7.49, 95% CI: 3.68 to 11.30; *P* < 0.001), and emotional functioning (72.52 ± 15.05 vs. 64.77 ± 14.31; MD 7.76, 95% CI: 3.34 to 12.18; *P* < 0.001). Symptom burden was lower in the D-PNP group, with reduced fatigue (26.86 ± 11.68 vs. 34.56 ± 13.45; MD −7.70, 95% CI: −11.49 to −3.90; *P* < 0.001) and dyspnea scores (22.35 ± 9.85 vs. 30.49 ± 11.46; MD −8.14, 95% CI: −11.36 to −4.92; *P* < 0.001).

Psychological outcomes favored the D-PNP group (Table 4). At 3 months, the D-PNP group showed lower anxiety scores (HADS-A: 5.25 ± 2.50 vs. 6.73 ± 2.96; *P* < 0.001) and depression scores (HADS-D: 4.58 ± 2.29 vs. 5.92 ± 2.81; *P* < 0.001). Clinically significant anxiety was less frequent in the D-PNP group at 3 months (12.6% vs. 25.9%; *P* = 0.027). Clinically significant depression was numerically lower in the D-PNP group (9.2% vs. 18.8%), but the between-group difference did not reach statistical significance (*P* = 0.068).

**TABLE 4:** Comparison of HADS scores and anxiety/depression prevalence between groups.

Parameter	Group	Baseline (T0)	1 month (T2)	3 months (T3)
HADS-A score (mean ± SD)	D-PNP	6.78 ± 3.21	6.12 ± 2.89*	5.23 ± 2.56*
	CNP	6.89 ± 3.12	7.45 ± 3.34	6.67 ± 3.12
HADS-D score (mean ± SD)	D-PNP	5.89 ± 2.87	5.34 ± 2.56*	4.56 ± 2.34*
	CNP	5.78 ± 2.76	6.56 ± 3.12	5.89 ± 2.89
Anxiety prevalence [*n* (%)]	D-PNP	21 (24.1)	16 (18.4)*	11 (12.6)*
	CNP	20 (23.5)	26 (30.6)	22 (25.9)
Depression prevalence [*n* (%)]	D-PNP	15 (17.2)	12 (13.8)*	8 (9.2)*
	CNP	14 (16.5)	19 (22.4)	16 (18.8)

*P < 0.05 compared with CNP group at the same time point. HADS-A, Hospital Anxiety and Depression Scale - Anxiety subscale; HADS-D, Hospital Anxiety and Depression Scale - Depression subscale. Clinically significant anxiety or depression defined as score ≥ 8.

Rehabilitation compliance was significantly higher in the D-PNP group (86.2% vs. 68.2% with good or excellent adherence; χ^2^ = 7.920, *P* = 0.005). In the D-PNP group, app-recorded exercise completion rate was 82.3% and daily symptom recording rate was 78.6%.

The 6MWD at 3 months was 438.67 ± 65.78 m in the D-PNP group versus 402.34 ± 61.45 m in the CNP group (mean difference: 36.33 m, 95% CI: 17.18-55.49 m; *P* < 0.001), representing 96.1% and 88.9% of baseline values, respectively.

Unplanned 30-day readmission occurred in 3 patients (3.4%) in the D-PNP group versus 9 patients (10.6%) in the CNP group (risk difference: −7.1%, 95% CI: −14.7% to + 0.5%; Fisher’s exact *P* = 0.079). This difference did not reach statistical significance at the conventional α = 0.05 threshold; we therefore interpret it as a directionally consistent trend with the primary PPC outcome rather than as an independently significant effect. Primary reasons for readmission included pneumonia (D-PNP: 1; CNP: 4), symptomatic pleural effusion (D-PNP: 1; CNP: 3), and worsening dyspnea (D-PNP: 1; CNP: 2). Overall patient satisfaction at 3 months was 94.3% in the D-PNP group compared with 81.2% in the CNP group (*P* = 0.009).

## Discussion

In this quasi-experimental study, D-PNP was associated with a lower postoperative pulmonary complication rate, faster pulmonary function recovery, better functional and psychological outcomes, and higher rehabilitation adherence than conventional nursing. The PPC finding remained directionally consistent and statistically supported across propensity-score matching and multivariable adjustment for measured baseline confounders. The non-randomized design precludes causal inference, and all between-group differences are reported as associations.

The 24.7% PPC rate in the CNP group falls within the 15–37% range reported in contemporary thoracic surgery cohorts (6), so the comparator reflects realistic standard-of-care rather than substandard practice. The PPC reduction observed in the D-PNP group is in line with prior trials of structured perioperative rehabilitation programs in thoracic surgery (21, 22), and the FEV_1_ recovery curve mirrors patterns reported after enhanced recovery and prehabilitation interventions following lung resection (5, 22). The 94.4% recovery of baseline FEV_1_ at 3 months in our D-PNP group is comparable to the upper range reported for hospital-supervised pulmonary rehabilitation. Our psychological findings are also consistent with a recent umbrella review of 78 systematic reviews showing that digital health interventions improve mental health outcomes in cancer populations (23).

The effects of digital interventions on respiratory readmission and recovery outcomes are heterogeneous across studies. A systematic review of 12 randomized trials of digital interventions for COPD readmission found that only four trials reported reductions in hospitalization; the authors attributed the variation to differences in intervention design, patient demographics, and disease severity (24). Two design choices distinguish D-PNP from interventions that have shown mixed or null results: rehabilitation prescription, alert thresholds, and follow-up cadence are tiered to an individual risk score rather than applied uniformly, and the platform supports bidirectional symptom communication coupled to nurse escalation actions rather than passive symptom logging. These user-centered and adaptive design features may partly account for the favorable outcomes observed in our cohort.

Several mechanisms may explain the observed differences. Risk stratification allowed targeted resource allocation, with high-risk patients receiving intensified monitoring and intervention.9 Real-time symptom monitoring with automated alerts shortened the time from symptom onset to clinical response (25). Risk-tiered exercise prescriptions adjusted the training stimulus to individual capacity, lowering the risk of both under- and over-exertion (21, 22). Gamification probably improved motivation and sustained engagement (10). Finally, the App’s chat and video channels gave patients a reliable route for clinical questions in the weeks after discharge, when anxiety about new symptoms is common.

The study has four notable strengths. The outcome panel spans physiological, functional, psychological, and behavioral domains. Retention reached 93.5% at 3 months, which limits attrition bias. Outcome assessors were blinded to group allocation, which lowers measurement bias for objective endpoints. The 3-month follow-up window covers both the early phase and the intermediate phase of postoperative recovery.

Seven limitations should be considered. (1) **Selection bias:** the non-randomized design relied on patient self-selection, which may have favored patients who were more health-conscious, technologically literate, or motivated toward recovery. Although measured baseline characteristics were balanced, unmeasured confounders such as health literacy, socioeconomic status, and family support could have influenced outcomes. (2) **Single-center design**: enrollment at one tertiary teaching hospital limits external validity for community or rural settings. (3) **Differential adherence measurement:** app-recorded data in D-PNP versus self-reported questionnaires in CNP introduces measurement bias, and the higher granularity of App data may exaggerate the true between-group adherence gap. (4) **Limited follow-up duration**: the 3-month window precludes assessment of long-term outcomes, including disease-free survival, durability of psychological benefits, and sustained engagement with the digital platform. (5) **Cost-effectiveness not evaluated**: implementation costs were not quantified, leaving open questions about scalability in resource-limited settings. (6) **Digital divide:** the inclusion requirement of smartphone capability excluded older patients with low digital literacy, who often carry the highest perioperative risk; the observed benefits may therefore not generalize to that subgroup. (7) **Hawthorne effect and contamination bias:** D-PNP patients had more frequent contact with the nursing team via the App, so part of the observed benefit may reflect increased attention rather than the digital tool itself. Additionally, both arms were cared for by the same nursing team, which may have produced contamination through unintentional transfer of D-PNP-derived practices to CNP patients—a bias that would attenuate, not inflate, the between-group difference.

The D-PNP model is a scalable way to extend specialized perioperative nursing beyond the hospital and to bridge the care discontinuity that follows discharge. Embedding risk stratification, symptom telemonitoring, and on-demand video follow-up into a single platform standardizes rehabilitation across heterogeneous patients and reduces the call-based workload for follow-up nurses. For institutions considering adoption, our experience suggests that successful implementation depends on upfront investment in interoperable mobile infrastructure, structured staff training, and clear escalation pathways for App-triggered alerts. Centers with high thoracic surgical volume may benefit most, because fixed development costs are amortized over a larger patient base.

Multi-center, adequately powered randomized controlled trials are needed to establish causal effects, ideally with stratification by surgical volume and urban–rural setting. Longer follow-up ( ≥ 12 months) should be incorporated to assess durability of pulmonary function gains and impact on cancer-specific outcomes. Formal cost-effectiveness and budget-impact analyses should accompany future efficacy studies to inform reimbursement and scaling decisions. Strategies to bring D-PNP benefits to patients excluded by the current digital threshold—simplified interfaces, family-caregiver–assisted models, or hybrid telephone-plus-App pathways—merit dedicated evaluation. Finally, the underlying risk stratification algorithm should be prospectively validated against external cohorts and iterated as additional data accrue.

## Conclusion

This quasi-experimental study suggests that a digital personalized nursing pathway is associated with reduced postoperative pulmonary complications, accelerated functional recovery, improved quality of life, better psychological outcomes, and enhanced rehabilitation adherence compared with conventional nursing care in patients undergoing surgery for non-small cell lung cancer. While these findings support the potential value of integrating digital health technologies into postoperative nursing care, confirmation through randomized controlled trials is warranted before widespread implementation.

## Data Availability

The raw data supporting the conclusions of this article will be made available by the authors, without undue reservation.
