# Supplementary material for: Effectiveness of digital personalized nursing pathway on postoperative rehabilitation in patients with non-small cell lung cancer
Source: Front Med (Lausanne). 2026 Jun 17;13:1803994. doi: 10.3389/fmed.2026.1803994 (PMC13318976; doi:10.3389/fmed.2026.1803994)
Supplement: Supplementary file 1 [file Data_Sheet_1.pdf]

TREND Statement Checklist

#	TREND item	Status	Location in manuscript
1	Information on how units were allocated to interventions; structured abstract; information on target population or study sample	Reported	**Title** explicitly identifies the design ("A Quasi-Experimental Study"). **Abstract** uses the structured Objectives / Methods / Results / Conclusion format and states group sizes, allocation period, target population (NSCLC patients undergoing surgical resection), and primary outcomes.

Title and Abstract

Introduction

#	TREND item	Status	Location in manuscript
2	Scientific background and explanation of rationale; theories used in designing the behavioral intervention	Reported	**Introduction**, paragraphs 1–4 (background on NSCLC, PPCs, mHealth opportunity); paragraph 5 provides the operational definition of the digital personalized nursing pathway (D-PNP) and its four-component conceptual model. The schematic in Supplementary Figure S1 makes the conceptual framework explicit.

Methods

#	TREND item	Status	Location in manuscript
3	Eligibility criteria for participants; method of recruitment; recruitment setting; settings and locations where data were collected	Reported	**Methods** **Participants** (eligibility / exclusion criteria) and **Methods** **Study Design, Setting, and Ethical Considerations** (single-center setting at Department of Pulmonary and Critical Care Medicine, Shanghai Changzheng Hospital; January 2022 to June 2024).
4	Details of interventions for each study condition (content, delivery method, unit, deliverer, setting, exposure dose and duration, time span, adherence-enhancing activities, language/literacy/cultural appropriateness)	Reported	**Methods** **Interventions**: full description of *Conventional Nursing Pathway (CNP)* and *Digital Personalized Nursing Pathway (D-PNP)* across four temporal phases (preoperative / inpatient / transition / 3-month home rehabilitation). The new *Risk Stratification Algorithm* subsection details the deterministic scoring rule, three-tier mapping, and Yellow/Red alert thresholds with co-occurrence and risk-tier modifier rules.
5	Specific objectives and hypotheses	Reported	**Introduction**, final paragraph; **Methods** **Sample Size Calculation** states the prespecified hypothesized PPC rates (28% CNP vs 12% D-PNP).

6	Clearly defined primary and secondary outcome measures; methods to collect data and to enhance measurement quality; psychometric properties of instruments	Reported	**Methods Outcome Measures** (primary: PPC per ESTS criteria, pulmonary function recovery; secondary: EORTC QLQ-C30/LC13, HADS, rehabilitation compliance, 6MWD, 30-day readmission, satisfaction). **Methods Data Acquisition and Preprocessing** documents the digital data pipeline, calibrated equipment (Jaeger Vyntus Spiro), single certified pulmonary technician, blinded research nurse, REDCap double-entry, and outlier rule.
7	How sample size was determined; interim analyses and stopping rules if applicable	Reported	**Methods Sample Size Calculation**: Cohen's $h = 0.41$; $\alpha = 0.10$ prespecified, power = 0.80; required 75 per group; targeted 90 per group with 20% attrition margin; 92 enrolled per group. Post-hoc power calculation reports achieved power at $\alpha = 0.05$ (0.72) and $\alpha = 0.10$ (0.81). No interim analysis was conducted.
8	Unit of assignment; method used to assign units to study conditions; details of restriction (blocking, stratification, minimization); methods to minimize bias due to non-randomization (matching)	Reported	**Methods Group Assignment** (allocation by patient preference and digital readiness). Bias mitigation by 1:1 propensity-score matching with caliper 0.20 SD on a logit propensity score is detailed in **Methods Statistical Analysis** and applied in **Results**

			**Sensitivity Analyses for Selection Bias**.
9	Whether participants, those administering the interventions, and those assessing the outcomes were blinded; if so, how blinding was accomplished and assessed	Reported	**Methods Quality Control**: single-blind design with outcome assessors blinded to group allocation; participants and nursing staff could not be blinded due to the nature of the digital intervention (acknowledged as a limitation).
10	Description of the smallest unit being analyzed to assess intervention effects; analytical method when the unit of analysis differs from the unit of assignment	Reported	The unit of assignment and the unit of analysis are both the individual patient. Stated implicitly throughout **Methods**; per-protocol analysis is the primary approach (**Methods Statistical Analysis**).
11	Statistical methods to compare groups for primary outcomes; additional analyses (subgroup, adjusted); methods for imputing missing data; statistical software	Reported	**Methods Statistical Analysis**: t-tests / Mann–Whitney U; Pearson chi-square / Fisher's exact; mixed-design repeated-measures ANOVA with Greenhouse–Geisser correction; per-protocol primary; complete-case analysis (missingness <2%); Cohen's *d*, RD with 95% CI (Wald), NNT; prespecified sensitivity analyses (PSM and multivariable logistic with parsimonious 8-covariate model; full 13-covariate model as exploratory robustness; E-value);

Results

#	TREND item	Status	Location in manuscript
12	Flow of participants through each stage of the study (diagram strongly recommended)	Reported	**Figure 1** flow diagram; expanded caption reports screening exclusions (4 concurrent malignancies, 2 cognitive impairment, 4 unable to use smartphone, 2 declined), allocation (D-PNP n=92, CNP n=92), follow-up attrition by reason, and per-protocol analysis cohort (D-PNP n=87, CNP n=85; completion 93.5%).
13	Dates defining periods of recruitment and follow-up	Reported	**Methods Study Design** and Figure 1 caption: enrolment January 2022–June 2024, follow-up extending 3 months post-surgery.
14	Baseline demographic and clinical characteristics of participants in each study condition; characteristics for those lost to follow-up vs retained; comparison between study sample and target population	Reported	**Table 1** (age, sex, BMI, smoking, comorbidities, TNM stage, surgical procedure, surgical approach, baseline FEV_1 and FEV_1% predicted in each group).
15	Data on study group equivalence at baseline and statistical methods used to control for baseline differences	Reported	**Table 1** P values; **Results** narrative confirms no statistically significant baseline differences. Where unmeasured imbalance might remain, propensity-score matching and multivariable logistic

			regression are reported in **Results Sensitivity Analyses for Selection Bias** (PSM: 79 matched pairs, all SMDs <0.10; OR 0.36, 95% CI 0.13–0.93, P=0.020; adjusted OR 0.33, 95% CI 0.13–0.77, P=0.013).
16	Number (denominator) included in each analysis; results in absolute numbers; intention-to-treat or per-protocol statement	Reported	Denominators reported per outcome in Tables 2–4 and inline (e.g., "9 of 87 D-PNP patients" for PPC). Per-protocol analysis declared as primary in **Methods Statistical Analysis**.
17	For each primary and secondary outcome, a summary of results for each study condition, with effect size and confidence interval (including null and negative findings)	Reported	**Results Primary Outcomes** (PPC: RD −14.4%, 95% CI −25.2% to −3.6%, NNT=7; FEV_1/FVC repeated-measures ANOVA Tables 3); **Results Secondary Outcomes** (QoL with mean differences and 95% CIs; HADS-A and HADS-D scores P<0.001 with depression prevalence flagged as P=0.068, not statistically significant; 6MWD MD 36.33 m, 95% CI 17.18–55.49; 30-day readmission Fisher's exact P=0.079 explicitly flagged as not reaching α=0.05).
18	Summary of additional analyses (subgroup, restricted), indicating prespecified vs exploratory	Reported	**Results Sensitivity Analyses for Selection Bias**: PSM (prespecified), parsimonious multivariable logistic (prespecified), full 13-covariate model (labelled exploratory),

			E-value (prespecified).
19	Summary of important adverse events or unintended effects in each study condition (with effect size estimates and confidence intervals)	Reported	PPC components (pneumonia, atelectasis, pleural effusion, prolonged air leak, respiratory failure) reported in **Table 2** with χ^2 and P values. 30-day readmission and reasons documented in **Results**. One non-study-related death recorded in the CNP attrition column (**Figure 1** caption). No App-related adverse events were observed.

Discussion

#	TREND item	Status	Location in manuscript
20	Interpretation taking into account study hypotheses, sources of bias, imprecision, multiplicative analyses, other limitations; mechanism by which intervention was intended to work; success of and barriers to implementation; research, programmatic, or policy implications	Reported	**Discussion** is structured into six IMRAD subsections: *Principal Findings*, *Comparison with Existing Literature*, *Potential Mechanisms*, *Strengths and Limitations* (4 strengths + 7 enumerated limitations with bias direction), *Clinical Implications*, *Future Directions*.
21	Generalizability of the findings, taking into account study population, intervention characteristics, follow-up, incentives, compliance rates, specific sites and settings	Reported	**Discussion Strengths and Limitations** items (2) single-centre design and (6) digital divide explicitly limit external validity; **Discussion Future Directions** specifies multi-centre RCT, ≥12 month follow-up, and strategies for digitally excluded populations.

22	General interpretation in the context of current evidence and current theory	Reported	**Discussion Comparison with Existing Literature** (PPC range 15–37% in contemporary cohorts; FEV_1 recovery comparable to hospital-supervised pulmonary rehabilitation upper range; psychological benefits aligned with Zhong et al. umbrella review of 78 systematic reviews; heterogeneity of digital interventions framed against Mishra et al. 2024 BMC Digital Health systematic review).
