# Supplementary figures and images for: Effectiveness of digital personalized nursing pathway on postoperative rehabilitation in patients with non-small cell lung cancer
Source: Front Med (Lausanne). 2026 Jun 17;13:1803994. doi: 10.3389/fmed.2026.1803994 (PMC13318976; doi:10.3389/fmed.2026.1803994)

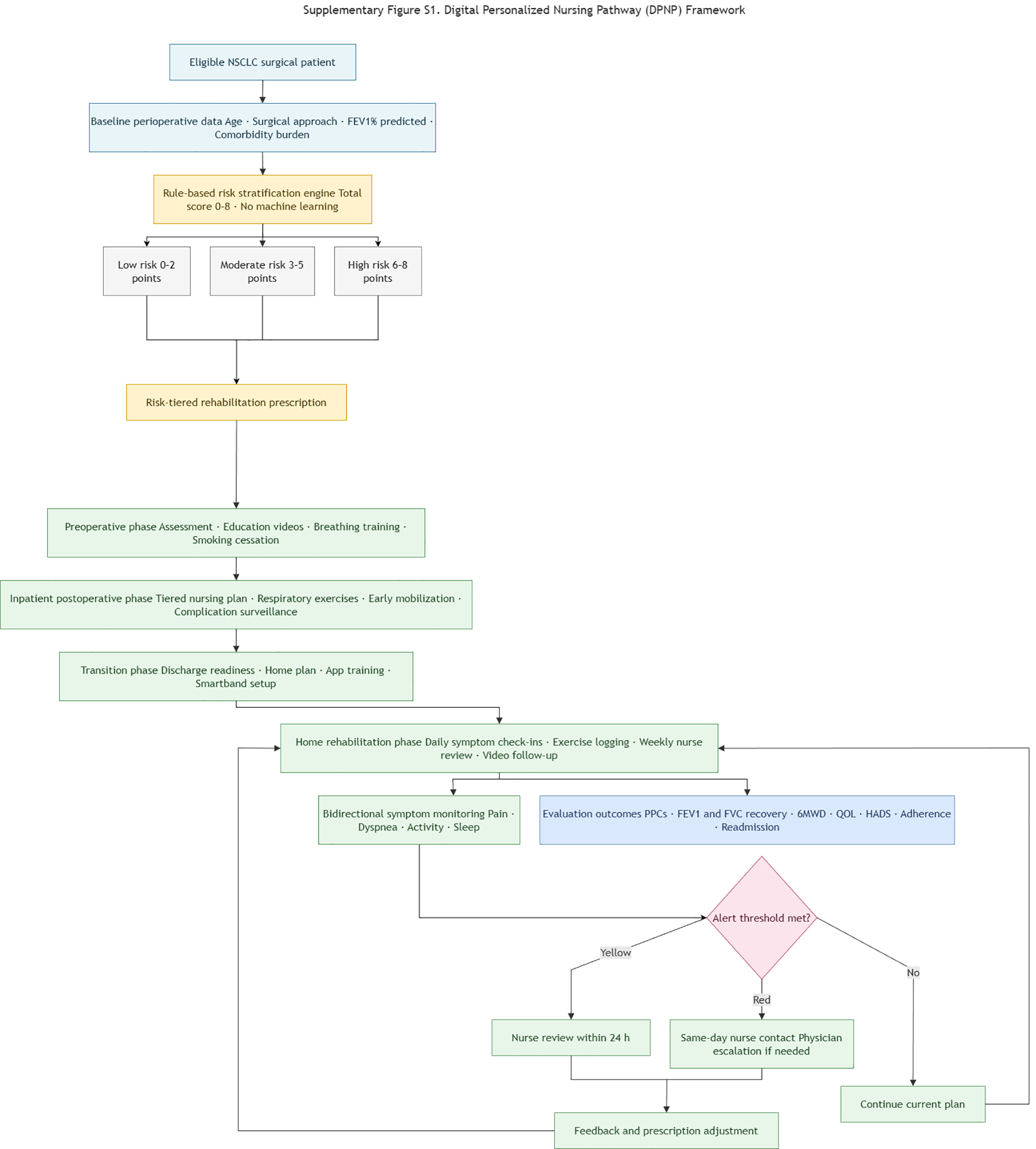

Supplement: Supplementary file 2 [file Image_1.png]
