# Supplementary material for: Effectiveness of digital personalized nursing pathway on postoperative rehabilitation in patients with non-small cell lung cancer
Source: Front Med (Lausanne). 2026 Jun 17;13:1803994. doi: 10.3389/fmed.2026.1803994 (PMC13318976; doi:10.3389/fmed.2026.1803994)
Supplement: Supplementary file 3 [file Table_1.docx]

**Supplementary Methods S1. Operational Details of the Digital Personalized Nursing Pathway**

**Conventional Nursing Pathway (CNP)**

Patients in the control group received standardized care according to institutional protocols, including: (1) Preoperative phase: routine health education, preoperative assessment, and psychological support; (2) Postoperative phase: vital signs monitoring, pain management, respiratory exercise guidance (incentive spirometry, diaphragmatic breathing), early mobilization, nutritional support, and complication surveillance; (3) Discharge phase: discharge education, provision of rehabilitation guidance booklet, and follow-up schedule; (4) Post-discharge follow-up: telephone follow-up at 1 week, 1 month, and 3 months post-discharge (approximately 10-15 minutes each), assessing recovery status and providing guidance. No digital tools were utilized in this group.

**Digital Personalized Nursing Pathway (D-PNP)**

In addition to standard care components, patients in the intervention group received a comprehensive digital intervention delivered through a purpose-built mobile health application ("LungRehab"). The platform comprised six integrated modules: (1) Patient information management with electronic health records; (2) Algorithmic risk stratification; (3) Personalized rehabilitation protocols with exercise prescriptions tailored to risk category; (4) Daily symptom monitoring (pain, dyspnea, activity level, sleep quality) with automated alerts when preset thresholds were exceeded; (5) Early warning system with automatic notifications to responsible nurses; and (6) Remote follow-up capabilities including video consultations and health education delivery.

**Risk Stratification Algorithm**

Each enrolled patient received an integer risk score (0-8) computed by the App from four perioperative domains, weighted equally to reflect the available evidence base for each component:

1. **Age**: 0 points if <60 years, 1 point if 60-69 years, 2 points if >=70 years.

2. **Surgical approach**: 0 points for video-assisted thoracoscopic surgery (VATS), 2 points for open thoracotomy.

3. **Baseline FEV1% predicted**: 0 points if >=80%, 1 point if 60-79%, 2 points if <60%.

4. **Comorbidity burden**: 0 points if none, 1 point for one or two of {hypertension, diabetes, chronic obstructive pulmonary disease, coronary heart disease}, and 2 points for three or more.

Total scores were mapped to three risk tiers: **low** (0-2 points), **moderate** (3-5 points), and **high** (6-8 points). The tier thresholds were derived from a pre-trial pilot cohort of 30 thoracic-surgery patients enrolled at our institution and refined by consensus of two senior thoracic surgeons and one pulmonary rehabilitation specialist. Each tier mapped onto a distinct rehabilitation prescription, including breathing-exercise frequency, ambulation targets, and symptom-alert thresholds.

**Symptom-Alert Rules**

Daily symptoms entered through the App were evaluated against two alert levels.

**Yellow alerts** (review at the next scheduled nurse touchpoint, <=24 h) were triggered when any of the following persisted for at least 1 day:

1. Numerical pain rating >=4.

2. Modified Medical Research Council (mMRC) dyspnea tier increased by >=1 from the patient's running 7-day baseline, or Borg CR-10 score >=4 at rest.

3. Step count below 50% of the personalized daily target for two consecutive days.

4. Sleep duration <5 h with quality rating <=2 for two consecutive nights.

**Red alerts** (same-day nurse contact, with physician escalation if symptoms persisted) were triggered by:

1. Pain rating >=7.

2. New pleuritic-character pain.

3. Borg CR-10 score >=7 at rest or new orthopnea.

4. Step count <2,000/day for three consecutive days.

5. Sleep duration <4 h for two consecutive nights.

Two concurrent Yellow alerts on the same calendar day were upgraded to Red. For patients in the high-risk tier, pain and Borg Yellow thresholds were tightened by one unit to allow earlier intervention. The algorithm was implemented in the App as a deterministic rule-based scoring engine; recomputation occurred whenever any input variable changed during the inpatient stay.

**Intervention Phases**

The D-PNP intervention was implemented across four phases:

1. **Preoperative phase**: electronic health assessment questionnaire completion, automated risk stratification report generation, and delivery of personalized preparatory video education (respiratory exercises, smoking cessation guidance, nutritional preparation).

2. **Inpatient postoperative phase**: risk-stratified nursing plans with intensified interventions for high-risk patients (increased respiratory exercise frequency, enhanced complication monitoring), plus real-time monitoring through app-recorded daily recovery data.

3. **Transition phase (1-2 days before discharge)**: discharge readiness assessment via App, generation of a personalized home rehabilitation plan, App usage training, and provision of a wearable activity tracker (smartband) for monitoring daily activity and sleep quality.

4. **Home rehabilitation phase (3 months)**: daily symptom self-assessment and exercise logging, dynamic protocol adjustment based on progress, weekly nurse review of patient data with individualized feedback, telephone or video follow-up within 24 hours for alert triggers, biweekly health education content delivery, and video follow-up assessments at 2 weeks, 1 month, and 3 months.
